# Lactoferrin: A Critical Player in Neonatal Host Defense

**DOI:** 10.3390/nu10091228

**Published:** 2018-09-04

**Authors:** Sucheta Telang

**Affiliations:** 1 Division of Neonatology, Department of Pediatrics, University of Louisville, Louisville, KY 40202, USA; 2 Division of Hematology/Oncology, Department of Medicine, James Graham Brown Cancer Center, University of Louisville, Louisville, KY 40202, USA

**Keywords:** lactoferrin, human milk, infection, immunity

## Abstract

Newborn infants are at a high risk for infection due to an under-developed immune system, and human milk has been shown to exhibit substantial anti-infective properties that serve to bolster neonatal defenses against multiple infections. Lactoferrin is the dominant whey protein in human milk and has been demonstrated to perform a wide array of antimicrobial and immunomodulatory functions and play a critical role in protecting the newborn infant from infection. This review summarizes data describing the structure and important functions performed by lactoferrin in protecting the neonate from infection and contributing to the maturation of the newborn innate and adaptive immune systems. We also briefly discuss clinical trials examining the utility of lactoferrin supplementation in the prevention of sepsis and necrotizing enterocolitis in newborn infants. The data reviewed provide rationale for the continuation of studies to examine the effects of lactoferrin administration on the prevention of sepsis in the neonate.

## 1. Introduction

The neonatal period is an exceptionally vulnerable period of life, during which term and preterm infants are at high risk for morbidity and mortality. According to recent data from the World Health Organization, 2.6 million neonates died globally in 2016 alone—accounting for 46% of the deaths under the age of five years [1]. Infections are responsible for approximately 36% of the deaths that occur in the newborn period [1], and there thus exists an urgent need for better strategies and approaches to improve neonatal outcomes worldwide.

The increased susceptibility of the newborn infant to infection is largely due to the immaturity of the neonatal immune system. Limited antigenic exposure in the predominantly sterile in utero environment is a dominant factor contributing to the underdevelopment of the adaptive immune response. Additional contributory factors are deficiencies in the cells responsible for adaptive immunity themselves—they are present in smaller numbers and show great variability in their adaptive responses [2]. As a result, to combat early infectious threats, newborn rely on their innate immune response, which is also not yet fully developed [3,4].

Neonatal deficiencies in immunity and host defense are compensated by several mechanisms. An early mechanism is the acquisition of antibodies passively transferred through the placenta from the mother [5]. Since this transfer occurs largely in the third trimester, the term infant is able to benefit from these antibodies but the preterm infant is unfortunately deprived of their protection.

A critical component of the armamentarium of the term and preterm neonate against infection is contributed by human milk. Human milk contains a wide array of bioactive proteins, growth factors, cells, and other constituents that modulate the development of a competent immune system to defend the term and preterm newborn against infections [6]. Of the bioactive factors present in human milk, lactoferrin has emerged as a key player that performs wide-ranging functions to directly and indirectly protect the neonate against infection.

## 2. Lactoferrin Distribution and Properties

Lactoferrin (or lactotransferrin, Lf) is a glycoprotein from the transferrin family of proteins. Lf was first identified in bovine milk by Sørensen and Sørensen in 1939 [7], then isolated from human and bovine milk by several investigators in 1960 [8,9,10]. Human Lf is a ~78 kDa glycoprotein which contains 691 amino acids and is expressed and secreted by epithelial cells in many exocrine secretions, including saliva, tears, and milk [11,12].

In human milk, Lf is the most abundant protein in the whey fraction, with a concentration varying from 1 gm/L to 7 gm/L (in colostrum) [12]. Multiple studies have evaluated Lf concentrations in colostrum and mature milk and in term and preterm milk. An early study that compared Lf levels between colostrum and mature milk in 30–32 week and >39 week neonates found trends towards higher initial Lf levels in the term infant group and higher sustained Lf levels in the preterm mature milk, but the differences did not reach significance [13]. A recent and comprehensive study has examined maternal milk samples from 24 week to term infants, and from birth to >10 days after birth, and found that Lf levels were highest in milk samples from mothers with infants <1400 g and that the levels varied significantly over time and with gestation [14]. Interestingly, the variation between samples within groups appeared fairly uniform, indicating that Lf concentrations in maternal milk at similar gestations may be relatively similar [14].

Lf levels are also sensitive to low and high temperatures. Studies (from our group) found that refrigeration of human milk samples (at 4 °C) for up to 5 days did not significantly lower Lf levels, but freezing (to −18 to −20 °C) decreased Lf dramatically to ~35% of the levels in fresh milk by 6 months, with a similarly significant decrease in its activity (by ~43%, measured by nitric oxide production) [15,16]. Heating also appears to decrease Lf levels, indicated by data showing that pasteurization (62.5 °C for 30 min, Holder method) significantly decreased the total protein (and thus presumably Lf) in human milk samples [17,18]. Further studies, in donor milk samples, showed an even more dramatic decrease (up to 88%) in Lf levels due to pasteurization [19]. This, when coupled with the freezing that these samples are exposed to, may indicate why donor milk has not shown the advantages of fresh maternal milk in terms of reduction in sepsis and necrotizing enterocolitis [18,19,20]. The detrimental effects of Holder pasteurization on immunological proteins in human milk have led to the active exploration of alternative methods to process donor human milk. Of these methods, exposure to 72 °C for 15 s (high temperature/short time or HTST pasteurization) has been demonstrated to preserve the integrity of Lf to a greater extent than the Holder method, although a significant decline in Lf relative to untreated milk is still noted [21,22,23,24]. Interestingly, studies have found that human Lf exposure to HTST conditions had only mild effects on its anti-bacterial activity [25], which may indicate that isolated and recombinant Lf may be less susceptible to temperature variations. Non-thermal alternatives to process donor milk are also under evaluation, such as high pressure processing, which has been shown to efficiently destroy microorganisms and allow greater retention of the immune components of human milk, including Lf [21,24]. A highly promising method that is currently under study is ultraviolet-C (UV-C) radiation. Recent data have indicated that UV-C radiation causes significant retention of Lf relative to Holder pasteurization and additionally induces greater resistance to bacterial infections in vivo [26,27,28]. In addition, these studies have described a technique to deliver UV-C radiation that has successfully overcome the limitations imposed by the high absorption coefficient of human milk [27]. These alternative processing methods will require extensive further investigation before reaching clinical application but certainly carry great promise.

The crystal structure of human Lf (hLf) was first solved in 1987 [29] and the protein has since been well described [30]. HLf contains two homologous lobes, each of which binds one ferric iron (Fe^3+^) with high affinity, making hLf a strong scavenger of iron. Lf is also able to retain bound iron down to a pH of ~3.5 [31] due to interactions between the 2 lobes, allowing it to be an effective anti-oxidant and bacteriostatic agent. Depending on its metal ion status, Lf can adopt either an iron-bound closed (holo-Lf) or a metal-free open conformation (apo-Lf)—both states have been demonstrated to perform functions in host defense. Lf additionally carries a high positive charge, with an isoelectric point of 9–10 that provides a high propensity for binding to negatively charged molecules on cell surfaces or in solution. Of particular importance are the basic residues at the N-terminus of Lf, at which proteolytic cleavage releases a potent antimicrobial peptide termed lactoferricin (Lfc) [32] that is highly exposed in both apo- and holo-Lf and may enable binding to bacterial cell membranes. A second peptide sequence, lactoferrampin, also has been identified as a major binding site with potential antibacterial properties. Additional data indicate that the glycan chains of Lf may mediate certain anti-bacterial and anti-viral activities as well [30].

Human Lf shares ~70% sequence homology with bovine Lf (bLf) [33], which has a molecular weight of ~76 kDa [34] and consists of 689 amino acids, and is both folded into N and C lobes and has antigenic determinants highly similar to its human counterpart [35]. Bovine Lf has a lower iron affinity than hLf, potentially due to altered interdomain interactions in its structure driven by the orientation and domains of its lobes and by its oligosaccharide units (particularly a glycan chain at Asn 545) [35,36]. Despite this difference, near-identical functions of human and bovine Lf [11] against multiple pathogenic organisms have been well documented [11]. Similar to human Lf, bovine Lf generates Lfc by cleavage at the cationic N-terminal region, which has been shown to cause a rapid loss of colony-forming capability [37]. Interestingly, the bovine Lf-generated Lfc was observed to have greater efficacy than human Lf against Gram-negative and Gram-positive bacteria [37]. Since bovine Lf is generally recognized as safe by the United States Food and Drug Administration (GRAS), it is easily available commercially and has therefore been widely used in vitro and in vivo for the examination of the various functions of this protein. In a recent study, commercial bLf added to infant formula was compared with hLf in an intestinal enterocyte model [38]. Commercial bLf was found to bind to the cells, be taken up by the human lactoferrin receptor, internalize, and promote proliferation and differentiation, indicating that it will likely exert bioactivities similar to hLf if supplemented in infant formula [38]. Several clinical trials examining the effects of bLf on infection have been conducted in preterm and term neonates, where bLf has been tolerated well (see Section 7). Bovine Lf-containing formula is also currently under active study in a clinical trial (NCT#02103205) evaluating the effects of the addition of bLf on the immune system, the microbiota composition, metabolomics, growth, body composition, and cognitive development.

The variable Lf levels in maternal milk likely indicate the evolving requirement for this protein with gestational and post-natal age, and these data form an important basis for the development of optimal strategies for infants who require supplementation. Although the susceptibility of Lf in milk to heat and cold may hamper the use of stored human milk for such strategies, the stability of isolated hLf and the similarities between hLf and bLf structure and function indicate the potential utility of these proteins in formula supplementation.

## 3. Direct Anti-Microbial Effects

**Figure 1:**
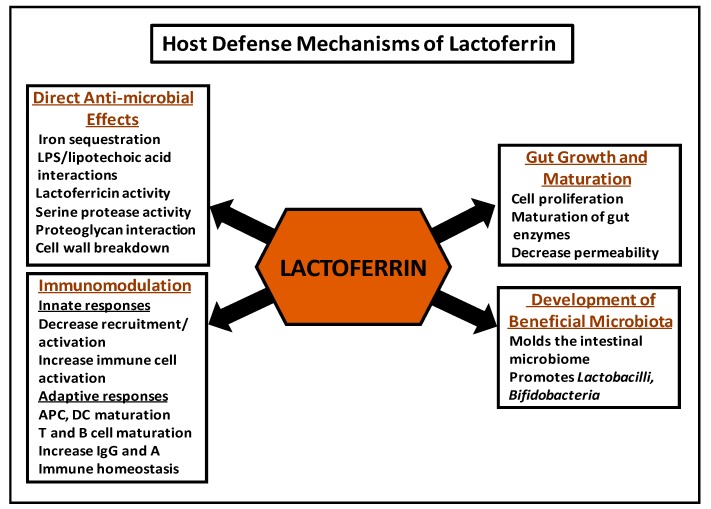
Functions of Lactoferrin in Neonatal Host Defense.

The anti-microbial effects attributed to Lf (Figure 1) were initially believed to be entirely due to the ability of unsaturated Lf to avidly bind iron and thereby cause bacteriostatic effects in iron-requiring pathogens. Early studies indicated that human milk and Lf purified from human milk had bacteriostatic effects on the growth of *E. coli* that were lost on saturation with iron [39]. These investigators went on to examine the effects of Lf against *E. coli* in vivo by gavage-feeding guinea pig pups with *E. coli*, and then either allowing the pups to suckle or feeding them with a milk substitute diet. They found that the suckled pups had substantially lower intestinal *E. coli* counts, interestingly with a corresponding increase in *Lactobacillus* numbers, and that the decrease in counts was reversed by feeding the pups hematin [39]. Iron-dependent anti-microbial effects of human and bovine Lf have been observed against a number of pathogens, including *S. mutans, V. cholerae*, and also *P. aeruginosa*, where iron chelation by Lf was found to stimulate a form of cell motility that inhibited biofilm formation by these bacteria [40,41,42,43,44].

Several studies demonstrate that, independent of its iron-binding capabilities, Lf is bactericidal to several pathogens [45,46] through interactions with the lipopolysaccharide (LPS) of Gram negative and the lipotechoic acid of Gram-positive bacteria [11]. In *E. coli*, Lf inhibits adherence and biofilm formation potentially by binding to lipid portions of the LPS layer, with a resultant increase in membrane permeability and disruption of virulence proteins anchored to the outer membrane [47]. These activities may be due to the action of Lfc—the peptide formed by the cleavage of Lf [32]. Further studies have determined another distinct anti-microbial function of the N-lobe of Lf due to the formation of a catalytic dyad by Ser259 and Lys73 that has a serine protease activity shown to successfully cleave and remove adherence elements of *H. influenzae*, thus attenuating its pathogenic potential [48,49]. An additional potential anti-bacterial mechanism has been proposed for Lf wherein it may enhance anoikis of infected enterocytes [50], but this activity requires significant further investigation. Taken together, these data indicate that the ability of Lf to affect bacterial attachment and invasion proteins may play a role in protecting suckling animals from infection by preventing the attachment and colonization of bacteria in the intestinal epithelium. In support of this hypothesis, studies have demonstrated that neonatal rodents pretreated with Lf had less bacteremia and less severe disease due to intestinal *E. coli* infection [51]. In addition, Lf has shown potent synergistic activity in killing Gram-negative bacteria in vitro with lysozyme—a second important component of the human milk whey fraction that is able to degrade bacterial membrane peptidoglycans. By binding LPS and removing it from the outer cell membrane, Lf allows lysozyme to access and degrade the inner membrane proteoglycans and kill the bacteria [52]. The bactericidal activity against Gram-positive bacteria appears to be caused by the same residues as with Gram-negative bacteria [53]. Of interest, a recent study examined the effects of *S. aureus* bacteremia in piglets pre-treated with dietary bovine Lf [54] and found that bLf pretreatment effectively reduced *S. aureus* systemic infection. BLf additionally decreased IL-10 and increased interferon-γ mRNA in these animals, indicating a type 1 T helper (Th1) immune response and, thus, effects on the innate and adaptive immunity of these animals. These results may explain some of the beneficial effects of bLf observed in preterm infants.

Lf also has direct inhibitory effects on viruses and other microbes. Against viruses, these effects may involve the attachment of Lf to surface proteoglycans, such as heparan sulfate, to which Lf has a high affinity through its N-terminus glycosaminoglycan-binding domains [55], thus blocking the entry of certain viruses, e.g., HSV. Other mechanisms may involve direct interactions of Lf with viral envelope proteins [56]. In fungi such as *Candida*, Lf has been shown to have effects as well, and was observed to cause cell wall perturbations, with the formation of surface blebs, swelling, and the collapse of the cell [57].

Variable responses to Lf-driven inhibition have been observed in different micro-organisms that are likely driven by differences in their iron requirement and their strategies to increase iron uptake and, additionally, by structural variations that may serve to limit direct access by Lf. As examples, several bacterial species have developed mechanisms to evade the iron-limiting effects of Lf. *Neisseria* and *Moraxella* species express specific Lf receptors that bind Lf to induce a conformational change in its structure and release iron into the bacteria [58]. Other micro-organisms have developed strategies to resist direct Lf-driven killing, such as *S. pneumoniae*, which binds Lf by pneumococcal surface protein A (PspA) and thereby evades the bactericidal effects of Lf [59], and *V. vulnificus*, which expresses a metalloprotease (Vvpe) that destroys Lf and facilitates the ability of the bacteria to invade the mucosa [60].

The studies indicate that further investigation into the anti-microbial functions of Lf is required. The examples described above notwithstanding, the available scientific evidence demonstrates the widespread inhibitory effects of Lf on the proliferation and survival of pathogenic micro-organisms—either by the sequestration of iron or direct activity on virulence factors—and strongly supports a protective role for Lf against infection in the newborn.

## 4. Immunomodulatory Functions of Lactoferrin

Lf plays a key role in neonatal host defense by modulating the innate and adaptive immune response of the neonate to infections (Figure 1). In addition, a growing body of evidence suggests that Lf facilitates mechanisms whereby adaptive immune changes may influence the innate immune system.

### 4.1. Mechanisms of Interaction of Lactoferrin with Immune Cells

The effects of Lf on immune cells are modulated by binding to a variety of targets. Among the most abundant are the glycosaminoglycans on membrane peptidoglycans [61], which are critical for the binding of many cytokines and factors, and it has been postulated that Lf may alter immune cell function by displacing these factors [62]. Other receptors described include lectins (e.g., TLR-4) which recognize the glycan chains of Lf, receptors recognizing the Lfc or N1 domain, intelectin-1 (found on enterocytes and immune cells), and nucleolin which may serve the additional function of transporting Lf to the nucleus [61,63]. All these receptors may potentially internalize Lf with downstream activation of signaling pathways e.g., the phosphoinositide 3-kinase (PI3K)/Akt and the mitogen activated protein kinase (MAPK)/extracellular signal-regulated kinase (ERK) signaling pathways, whereby Lf has been shown to activate cell cycle progression, proliferation, and downstream cellular responses [64,65,66] or, following nuclear localization, the NFkB pathway [61,67]. In addition, Lf has been found to bind to a receptor on (transformed) hematopoietic cells and translocate to the nucleus, leading to transcriptional activation with downstream effects [68,69].

### 4.2. Innate Immune Effects of Lactoferrin

The effects of Lf on the innate immune response are related in part to its ability to bind to conserved structures, termed pathogen-associated molecular patterns (PAMPs), present on pathogens (e.g., LPS on Gram-negative bacteria and peptidoglycans on Gram-positive bacteria). PAMPs are recognized by pattern recognition receptors or PRRs such as Toll-like receptors (TLRs) [70], that are critical for the activation of innate immunity. TLR4 has been demonstrated to bind and transfer LPS, with the assistance of the transfer molecule LPS-binding protein (LBP), to CD14. CD14 is a glycosylphosphatidylinositol-anchored membrane protein present on myeloid cells which leads to their activation and the release of pro-inflammatory cytokines e.g., TNF-α, IL-6, and IL-1β [71,72]. Lf is demonstrated to bind to several PAMPs, including LPS, and thereby compete with LBP to inhibit the release of pro-inflammatory cytokines [62,73]. Lf may also modulate recruitment of immune cells by interfering with the expression of endothelial cell adhesion molecules required for the recruitment of these cells to sites of inflammation as shown by data indicating that the interference of Lf in the LPS-CD14 interaction may inhibit the expression of E-selectin, ICAM-1, and IL-8 by human umbilical vein endothelial cells (HUVECs) [62,74]. Lf may cause further suppressive effects on immune cells by binding to other molecular cell surface targets, as evident by its function in competing with the chemokine IL-8 for binding to endothelial cell proteoglycans to inhibit the activation and recruitment of leukocytes to sites of inflammation [74].

Lf is also capable of enhancing the activation of immune cells. Following bacterial invasion, LPS binds to TLR4 on sentinel cells to cause the release of potent cytokines including TNF-α, IL-1β, and IL-6 [62,75]. These molecules will activate and modify the permeability of endothelial cells to allow the passage of complement and antibodies and recruit neutrophils to the site of inflammation. Activated neutrophils will release Lf from their secondary granules to exert its direct microbicidal effects [62]. Lf may also enhance the cytotoxic functions of NK and lymphokine-activated killer cells, potentially through binding to RNA and DNA [76].

Promotion of lytic cell activity is a key role played by Lf. Lf receptors are found on macrophages [77] and Lf is shown to activate macrophages to release pro-inflammatory molecules e.g., TNF-α, IL-8, and nitric oxide [15,78] and to increase their phagocytic activity when infected [79]. Lf is also expressed on the membranes of resting PMNs and may enable interaction between Lf-bound microbes and PMNs [80]. Bovine Lf was noted to increase phagocytic killing of *S. aureus*—potentially by activation of the alternate complement pathway by its Lfc domain [81,82].

### 4.3. Effects on Adaptive Immune Responses

Lf plays an important immunomodulatory role in activation and antigen presentation by antigen-presenting cells (APCs) and in their functions in the adaptive immune response by affecting T cell development. Macrophages function as APCs to stimulate the development of antigen-specific CD4+ T cells, and Lf enhances their ability to function as APCs by stimulating the production of cytokines, such as IL-12, responsible for modulating development of Th1 cells [83,84].

Lf also assists in the maturation of dendritic cells (DCs)—by enhancing their release of IL-8 and CXCL10, decreasing antigen internalization, increasing their capacity to trigger proliferation and release IFN-γ in the presence of allogeneic human T cells, and to prime naïve T cells in response to several antigenic stimuli [85]. Recent studies indicate that Lf may function similarly to an alarmin to promote the activation of APCs and antigen-specific immune responses [86,87]. These studies demonstrate that, similar to the previous study, Lf is able to chemoattract and cause the maturation of monocyte-derived DCs, and also stimulate the production of pro-inflammatory cytokines. Lf additionally may prompt Th1 polarized antigen-specific immune responses in immunized mice and the recruitment of macrophages and neutrophils when injected into the mouse peritoneal cavity [86].

More recent data indicate a role for Lf in immune homeostasis as well. Studies indicate that DCs differentiated in the presence of Lf showed decreased responsiveness towards TLR ligands [88,89] and reduced cytokine production demonstrating a potential role for Lf in immune homeostasis. These results indicate a potent anti-inflammatory function for Lf by skewing monocyte differentiation into DCs with impaired capacity for activation and for promotion of Th1 responses and may represent a strategy to block excessive DC activation upon TLR-induced inflammation, adding further evidence for a critical role of Lf in directing host immune function.

Lf has been shown to modulate the production of pro-inflammatory cytokines, such as TNF-α, IL-1β, and IL-6, from leukocyte populations, which may be increased or decreased depending on the condition recognized by the immune system. In addition, Lf may increase the production of IL-12 by APCs when presented with pathogens. IL-12 enhances IFN-γ production and proliferation, augments cytotoxic activity of lymphocytes responsible for innate (NK cells) and adaptive (CD4+and CD8+ T-cells) immunity, and is a major driver of Th1 cell development [83,84].

Lf also influences T and B lymphocyte maturation. Lf is able to bind to surface receptors and be internalized by human Jurkat lymphoblastic T cells [90], where it accelerates T cell maturation by induction of CD4 via activation of the MAPK pathway [91]. Human milk-derived Lf is observed to cause maturation of CD4^−^CD8^−^ murine T-cells, with a preference towards expression of CD4 [92]. When administered orally, Lf has the ability to restore the host T cell compartment, evident by an increase in splenic cellularity and enrichment of CD3+ CD4+ T cells, and suggesting a possible role for Lf in the reconstitution of the cellular immune response [93]. As noted with other cell types, Lf also appears to exert anti-inflammatory effects. The addition of Lf to mitogen-activated T-cells decreases overall cytokine production demonstrated by the decreased production of IFN-γ and IL-2 by ConA-stimulated murine splenocytes cultured with Lf [94]. Similarly, Lf is able to promote the maturation of immature B lymphocytes, shown by an increase in surface Ig D and complement receptor expression. In addition, Lf was shown to enable B cells from normal newborn and adult immunodeficient mice to present antigen to an antigen-specific T-helper type 2 (Th2) cell line [95]. Orally administered Lf has been demonstrated to increase the pool of CD4+ T cells, immunoglobulin levels (G and A), as well as proliferation in the Peyer’s patches of the intestine, suggesting that Lf may act as an immunostimulatory factor on the mucosal immune system [96,97,98]. In addition, in a chemotherapy-induced immune suppression murine model, Lf administered intraperitoneally was able to decrease the suppression of antibody forming cells and facilitate the restoration of the immune response [99].

Taken together, these studies illustrate the multiple activities performed by Lf to modulate the nascent neonatal immune system and highlight the importance of this protein in the development of a mature immune response. The growing body of scientific evidence suggests that the effects of Lf vary depending on the threat faced by the immune system and thereby emphasizes the importance of this glycoprotein in the protection of the newborn from infection.

## 5. Effects of Lactoferrin on the Development of Beneficial Microbiota

The bacterial flora colonizing human milk fed infants have been demonstrated to be different from those of formula-fed infants. Higher concentrations of *Lactobacillus* and *Bifidobacteria* species are observed with comparatively fewer bacteria with high pathogenic potential e.g., *E. coli*, *Campylobacter*, and *Bacteroides* [100]. In vitro studies have demonstrated that Lf from human and bovine milk promotes the growth of intestinal bifidobacteria without the requirement for binding of the Lf molecule to the bacterial cell surface or a dependence on the acquisition and utilization of iron [101]. Bifidogenic peptides have been isolated from human milk (derived from hLf) that demonstrate strong bifidogenic effects on several bifidobacterial species (*B. bifidum*, *B. breve*, and *B. longum)* and are resistant to digestive enzymes [102,103]. The importance of Lf in the development of beneficial bacteria is underscored by data from breastfed term and preterm infants, showing high fecal Lf levels and a significant association of bifidobacteria and lactobacilli with fecal Lf levels on day three of life, suggesting that Lf may be a key factor in the initiation, development, and composition of the neonatal gut microbiota [104]. These data indicate that Lf is a tremendously important influence on the development of the intestinal microbiome (Figure 1). The importance of this function of Lf is magnified in critically ill and hospitalized term and preterm infants who are at risk for colonization and infection with highly pathogenic bacteria [105], and where Lf administration may be able to play a critical role in decreasing invasive infection and necrotizing enterocolitis. Interestingly, recent studies have examined the effect of Lf on probiotic bacterial growth in vitro and found that both hLf and bLf may retard the growth of certain bifidobacteria [106,107]. In view of the importance of the development of beneficial gut bacteria and ongoing clinical examination of Lf supplementation with probiotics, the further delineation of the precise effects of Lf in probacterial growth is a critical avenue of investigation.

## 6. Effects on Gut Growth and Maturation

Lf has been found to directly stimulate intestinal growth and proliferation [108,109] (Figure 1). Studies conducted on Caco-2 (transformed) enterocytes in vitro have found that exposure to high Lf concentrations led to a dose-dependent increase in cell proliferation, while low Lf levels stimulated intestinal cell differentiation [108]. These data suggest that Lf may actively modulate enterocyte growth and development in vivo due to variations in its concentration from colostrum to mature milk, in addition to its stimulatory effects on intestinal enzyme maturation [108]. Of interest, these studies found that bLf was a more potent effector of growth than hLf, which provides rationale for its supplementation in infant formula [108]. Beneficial effects of bLf administration were also noted in vivo. Neonatal piglets fed formula that contained physiological levels of bLf relative to controls fed low bLf showed an increase in intestinal cellular proliferation and, additionally, increased β-catenin levels, indicating a potential role for Wnt signaling in gut proliferation [110]. Other studies have observed that Lf is taken up by enterocytes via the Lf receptor and stimulates enterocyte proliferation through the Ras-MAPK pathway [68], the strong mitogenic effect of which also may drive the rapid development of the intestinal mucosa in newborns fed maternal milk. An additional possible function for Lf in intestinal maturation is in regulation of gut permeability. In its support, studies have shown that preterm infants fed maternal milk had decreased gastrointestinal permeability relative to formula-fed controls, which indicates a potential role for components of human milk in intestinal maturation [111]. This mechanism of action will require further examination in the newborn population.

The functions performed by Lf in the growth and maturation of the gut are critical for the development and maintenance of the intestinal barrier to infection. The breakdown of this barrier may expose the newborn to potentially highly pathogenic bacteria. These findings therefore support the importance of early and continued exposure of the newborn gut to Lf in human milk or as a supplement in formula. 

## 7. Examination for Clinical Efficacy of Lf in Neonates

An overwhelming body of experimental evidence supports the beneficial anti-infective properties of Lf, providing strong rationale for its use against infection in newborn infants.

Based on the significant anti-microbial and immunomodulatory effects caused by Lf, this protein may be particularly useful in host defense in critically ill and very low birth weight (VLBW) neonates. VLBW infants carry an enhanced risk for bacterial sepsis and potentially devastating sequelae [112] and are frequently unable to tolerate feeds, thus depriving them of the protective benefits of maternal milk. Based on this rationale, several studies have examined the efficacy of Lf supplementation against sepsis [113] in the neonatal period. An early study where healthy, formula-fed infants (≥34 weeks gestation and ≤4 weeks old) were fed formula supplemented with bovine Lf vs. cow milk-based formula and followed for 12 months found significantly fewer lower respiratory tract illnesses in the Lf-fed group [114] (Table 1). In 2009, Manzoni’s group performed a multicenter, double-blinded, placebo-controlled, randomized trial in VLBW infants (<1500 g) comparing administration of bLf alone or in combination with *Lactobacillus rhamnosus* GG (LGG) to placebo [115]. They found significantly lower invasive infections in the treatment groups, with an effect on infection-related mortality (0% for bLf and 0.7% for bLf plus LGG, vs. 4.8% for placebo). A follow-up study from the same group in 2014 found that bLf supplementation alone or in combination with LGG significantly reduced the incidence of ≥stage 2 necrotizing enterocolitis (NEC), and of death-and/or ≥stage 2 NEC in VLBW neonates [116]. Apart from these, several other studies (Table 1) have also examined bovine Lf and found that treatment with bLf led to a reduction in infection in both VLBW and 500–2500 g neonates [117,118]. Importantly, none of these investigations noted any adverse effects or intolerance with bovine Lf. BLf has also been evaluated in a recent study that confirmed that it was well tolerated [119]. Several other studies examining the efficacy of bovine Lf are currently underway [113]. Of note, a multicenter trial of enteral bovine Lf in 2200 <32 week infants (the ELFIN trial UK) that has recently completed recruitment, will primarily evaluate effects on late onset invasive infection but also mortality, NEC, and several later sequelae [120]. The results of this large trial may serve to further validate the utility of bovine Lf supplementation in this vulnerable population.

Multiple in vitro and animal studies have demonstrated potent anti-microbial and immunomodulatory effects with Lf isolated from human milk. A recombinant human lactoferrin, generated in *Aspergillus oryzae,* was demonstrated to have an amino acid structure and functions highly similar to the human milk molecule [121]. Based on this expression system, commercial amounts of this protein were generated, leading to the development of a clinical candidate (talactoferrin) that differs from the native human protein in its glycosylation due to the fungal expression system but is otherwise unchanged [122,123]. Studies have demonstrated that talactoferrin is well tolerated in adult patients [124]. A single multicenter trial was conducted using talactoferrin in 750–1500 g neonates, which examined 120 infants and showed a trend towards decreased infectious morbidity but did not achieve statistical significance [125] (Table 1). Further trials with this protein, however, currently appear to be on hold following recent data showing no benefit in a trial in adult ICU patients [126].

Based on the pre-clinical data, the potential benefits of Lf supplementation are clear—with strong evidence supporting its direct anti-microbial and immune-boosting properties and effects on gut proliferation, maturation, and the development of beneficial bacteria. The clinical studies done thus far have shown uniformly positive results that have reached statistical significance in certain studies (Table 1). Based on the clinical data, early commencement of Lf may be associated with greater clinical benefits, demonstrated by examining study results from Ochoa et al. (Lf started with enteral feeds at 4 ± 1.4 days [118]), Akin et al. (with feeds at 20 mL/kg/day [117]), and Manzoni et al. (at <72 h [115]). Early supplementation may mimic the higher Lf in human colostrum and, as shown in vitro, may allow for early gut proliferation. The addition of a probiotic [115,116] appeared to substantially improve outcomes and should be further explored. However, as indicated by recent in vitro data described above [106,107], the administration of Lf in conjunction with a probiotic requires further careful study. Additionally, the use of a standard dose of Lf for all patients may not be optimal for delivering adequate concentrations of Lf to each patient and weight-based dosing regimens should be evaluated for clinical efficacy. Last, although the results with recombinant human Lf were not significant, the use of a human Lf might be revisited in the future.

## 8. Conclusions

Taken together, the experimental and pre-clinical studies examining the functions of Lf present overwhelming evidence, supporting a pivotal role for this multifaceted glycoprotein in preventing infection, in immunomodulation, and bolstering host defense. Many questions remain to be answered regarding the function of this glycoprotein at the molecular level and the extent of direct and immune modulatory effects caused by supplementation of Lf in the diet. Several of these questions are best addressed by in vivo studies in patients. These are challenging studies, particularly as they are targeted towards the critical VLBW infant. However, the clinical data obtained thus far have been promising and certainly support the utility of continuation of studies to examine the effects of Lf supplementation on modulating the immune response and decreasing life-threatening infections in the highly vulnerable neonatal population. Several studies are currently underway, and their results will serve to clarify the benefits of Lf supplementation in the diet of the term and preterm infant, and potentially pave the way to using Lf in the clinical setting.

## Figures and Tables

**Table 1:** Clinical Studies of Lactoferrin in Neonates. The *n* values denote the number of patients in the treatment groups. Significant study outcomes are in bold type. LOS, Late-onset sepsis.

Year	Study Population	Study Design	Lf Type	Outcomes	Investigator, Site
2007	Neonates ≥34 weeks, ≤4 weeks of life (*n* = 26)	Formula + Lf (850 mg/L) vs. cow—milk formula + Lf (102 mg/L) (≤4 weeks–12 months)	Bovine	**Lower incidence of lower respiratory tract infections**	King, USA
2009, 2012	VLBW Neonates <1500 g (Lf, *n* = 153, Lf +LGG, *n* = 151)	Lf (100 mg/day) ± LGG vs. placebo, 0–30 days (0–45 days for <1000g at birth)	Bovine	**Lower incidence of first LOS episode (in Lf ± LGG)** **Lower incidence of *Candida* LOS**	Manzoni, Italy
2014	VLBW neonates <1500 g (Lf, *n* = 247, Lf + LGG, *n* = 238)	Lf (100 mg/day) ± LGG vs. placebo, 0–30 days (0–45 days for <1000 g at birth)	Bovine	**Reduced incidence of ≥stage 2 NEC and of death and/or ≥stage 2 NEC**	Manzoni, Italy and New Zealand
2014	VLBW neonates, <1500 g or <32 weeks (*n* = 25)	Lf (200 mg/day) vs. placebo, through hospitalization period	Bovine	Decreased nosocomial sepsis episodes	Akin, Turkey
2015	Neonates, 500–2500 g (*n* = 95)	200 mg/kg/day vs. placebo from 2–28 days	Bovine	Sepsis less frequent in Lf group (Primary outcome: incidence of LOS, no statistical significance but CI suggestive of effect)	Ochoa, Peru
2015	Neonates <2000 g (*n* = 65)	Lf (80–140 mg/kg/day) vs. placebo from 1–28 days	Bovine	**Lower incidence of first LOS episode, reduction in sepsis-attributable mortality**	Kaur, India
2016	Neonates 750–1500 g (*n* = 60)	Lf (150 mg/kg q12h) vs. placebo from 1–28 days	Human	Trend towards decreased infectious morbidities (primary outcomes: bacteremia, NEC pneumonia, UTI, meningitis)	Sherman, USA
2016	Neonates <32 weeks (*n* = 40)	Lf (100 mg/day) vs. placebo, until 36 weeks PMA or discharge	Bovine	No difference in feeding tolerance	Barrington, Canada
